# Ruthenium Complex Improves the Endothelial Function in Aortic Rings From Hypertensive Rats

**DOI:** 10.5935/abc.20170090

**Published:** 2017-08

**Authors:** Izabela Pereira Vatanabe, Carla Nascimento dos Santos Rodrigues, Tereza Cristina Buzinari, Thiago Francisco de Moraes, Roberto Santana da Silva, Gerson Jhonatan Rodrigues

**Affiliations:** 1 Universidade Federal de São Carlos (UFSCar), São Paulo, SP - Brazil; 2 Universidade de São Paulo (USP), Ribeirão Preto, SP - Brazil

**Keywords:** Rats, Hypertension, Renal, Ruthenium, Endothelium / physiopathology, Nitric Oxide

## Abstract

**Background:**

The endothelium is a monolayer of cells that extends on the vascular inner surface, responsible for the modulation of vascular tone. By means of the release of nitric oxide (NO), the endothelium has an important protective function against cardiovascular diseases.

**Objective:**

Verify if cis- [Ru(bpy)_2_(NO_2_)(NO)](PF_6_)_2_ (BPY) improves endothelial function and the sensibility of conductance (aorta) and resistance (coronary) to vascular relaxation induced by BPY.

**Methods:**

Normotensive (2K) and hypertensive (2K-1C) Wistar rats were used. For vascular reactivity study, thoracic aortas were isolated, rings with intact endothelium were incubated with: BPY(0.01 to10 *µ*M) and concentration effect curves to acetylcholine were performed. In addition, cumulative concentration curves were performed to BPY (1.0 nM to 0.1 *µ*M) in aortic and coronary rings, with intact and denuded endothelium.

**Results:**

In aorta from 2K-1C animals, the treatment with BPY 0.1*µ*M increased the potency of acetylcholine-induced relaxation and it was able to revert the endothelial dysfunction. The presence of the endothelium did not modify the effect of BPY in inducing the relaxation in aortas from 2K and 2K-1C rats. In coronary, the endothelium potentiated the vasodilator effect of BPY in vessels from 2K and 2K-1C rats.

**Conclusion:**

Our results suggest that 0.1 *µ*M of BPY is able to normalize the relaxation endothelium dependent in hypertensive rats, and the compound BPY induces relaxation in aortic from normotensive and hypertensive rats with the same potency. The endothelium potentiate the relaxation effect induced by BPY in coronary from normotensive and hypertensive rats, with lower effect on coronary from hypertensive rats.

## Introdution

Endothelial dysfunction is characterized mainly by decreasing the ability of endothelial cells to release nitric oxide (NO),^1^ and it has been associated with hypertension as well as other cardiovascular diseases, furthermore, it includes release and superoxide anion (O_2_^-^) increased bioavailability generating to peroxinitrite (ONOO^-^) join reaction with NO. This reaction is present in dysfunctional endothelial cells 2K-1C animals, due to the current Angiotensina II increase.^2^

NO is involved in diverse pathophysiological process that encourages the emergence of researches about drugs that can be able to modulate NO concentration for therapeutic purpose,^3^ including NO donors.

On preliminary results, we have observed that the ruthenium complex *cis-*[Ru(H-dcbpy)_2_(Cl)(NO)] (dcbpy) improved the relaxation endothelium dependent induced by acetylcholine in aortic rings from hypertensive rats^4^. This compound also is able to induce relaxation by NO release in higher concentration, and the improvement in endothelial function was attributed to inactivation of O_2_^-^.^4^

The NO donors are pharmacologically active substances that release NO. The NO donors most widely used in medical practice are organic and inorganic nitrates, nitroglycerine and sodium nitroprusside, respectively. However prolonged treatment with these drugs have induced adverse effects, such as intolerance, endothelial dysfunction, release of toxic compounds, reflex tachycardia and other adverse effects that are limiting factors to the use of these NO donors.^5-8^

Thus, the macrocyclic nitrosyl ruthenium complexes are being studied as NO donors, ^9-14^ which are attractive because they have active forms that are stable and have low toxicity under physiological conditions.^10,12,13^ Another important feature displayed by these compounds is the sustained release of NO, as we noted in prolonged hypotensive effect generated in hypertensive animals ^15,16^ and that was also observed in studies of release kinetics NO *in vitro.*^17,18^

Exogenous NO donors agents based on ruthenium-derived metal nitrosyl complexes have been developed as strategy to reduce side effects and cytotoxicity. They have not displayed any toxic effects and they are able to induce vascular relaxation and decrease blood pressure in normotensive and hypertensive rats^15,19^ being the cis- [Ru(bpy)_2_ (NO_2_)(NO)] (PF_6_)_2_ (BPY) able to induce aortic relaxation and decrease blood pressure in normotensive rats.^20^

Thus, drugs in which the center of the metal is ruthenium, as BPY, have good clinical application, especially considering that the low toxicity of the metal ion is similar to the physical and chemical properties present in the iron metal ion.^21^ The body can protect from the effects caused by excess of iron ions with the formation of transferrin and albumin, therefore it is believed that the mechanism of protection against the toxicity of ruthenium would be the same.^21,22^ Thus, based on literature existing surrounding this issue, it appears that the BPY is more attractive to present active form under physiological conditions predicting a good future clinical application.^11-13^

## Objective

This study was made to evaluate if BPY improves endothelial function, and the sensibility of conductance (aorta) and resistance (coronary) to vascular relaxation induced by BPY.

## Methods

Materials used (Drugs and chemicals), Acetylcholine (Ach) and phenylephrine (Phe) were purchased from Sigma-Aldrich (St.Louis, MO, USA); Compound cis- [Ru(bpy)_2_ (NO_2_)(NO)] (PF_6_)_2_ (BPY) was synthesized by a partner in University of Pharmaceutical Sciences of Ribeirão Preto.

### Experimental animals

Male Wistar rats were used weighing between 180-200 grams. The animals were maintained on a standard diet with a 12 h cycle light/dark and free access to food (standard diet) and water. The animals were anaesthetized with Tribromoethanol (2.5 mg/kg, ip) after a midline laparotomy a silver clip with an internal diameter of 0.20 mm was placed around the left renal artery as previously described for 2K-1C by Goldblatt et al.^23^, where only one renal artery is restricted to reduce chronic renal perfusion. Normotensive two-kidney rats (2K, n = 6) were only submitted to laparotomy. Systolic blood pressure (SBP) was measured by a method of indirect tail plethysmography (MLT125R pulse pressure transducer/Cuss coupled to PowerLab 4/S-digital converter; AD Instruments Pty Ltd, Castle Hill, Australia) in animals not anaesthetized. The animal were considered hypertensive when systolic blood pressure was greater than 160 mmHg six weeks after surgery.

### Ethical aspects

Experimental protocols followed standards and policies of Animal Care and Use Committee of the Federal University of São Carlos (CEUA: 012/2013).

### Vascular reactivity study

Six weeks after surgery, rats were killed by decapitation and the thoracic aorta or coronary were dissected, cut into rings and placed in bath chambers containing Krebs solution at 37°C, pH 7.4, continuously bubbled with 95% O_2_ and 5% CO_2_, in an isometric myograph (Mulvany-Halpern-model 610 DMT-USA, Marietta, GA) and recorded by a PowerLab8/SP data acquisition system (ADInstruments Pty Ltd., Colorado Springs, CO).

Endothelial integrity was assessed by the degree of relaxation induced by 1 *µ*mol/L acetylcholine after contraction of the aortic ring by phenylephrine (0.1 *µ*mol/l). The ring was discarded if relaxation with acetylcholine was lower than 80% in 2K and 60% in 2K-1C rat aortas. After the endothelial integrity test, aortic rings were pre-contracted with phenylephrine (0.1 *µ*M) and then were constructed concentration-effect curves to acetylcholine (0,01 *µ*M to 10 *µ*M) and BPY (1,0 nM to 0.1 *µ*M), similarly in coronary artery rings, with and without intact endothelium, pre-contracted contractile agent (serotonin 10 *µ*M) cumulative concentration curves were performed for the purpose BPY compound.

Aortic rings from 2K and 2K-1C were treated for 30 min with BPY (at concentrations: 0.1 *µ*M) or PBS (control). The concentration of BPY chosen (0.1 *µ*M) is close to EC_50_. After incubation, aortic rings were washed three times to remove drugs, pre-contracted and concentration-effect curves to acetylcholine were constructed. The potency values (pD2) and maximum relaxant effect (ME) were analyzed. The curves concentration effect for BPY were realized without previous incubation.^29^

### Statistical analysis

Normality of distribution was checked with the Kolmogorov-Sminorv test, differences in means were compared by ANOVA. When significance was indicated, a Newman-Keuls post hoc analysis was used with statistical significance set at p < 0.05 (Software Prisma 3.0, Graphpad Software Inc, La Jolla, CA, USA). Data are expressed as mean ± S.D.

To calculate the sample size was followed the statistical formula for the calculation of the sample in an infinite population. In preclinical studies, we found that the standard deviation in the power of relaxation induced by acetylcholine in normotensive rat arteries was 0.31. We consider a tolerable sampling error of 0.25, thus define the size of the sample used in accordance with the formula: n = (1.96X0.31/0.25) 2 = 5.9 animals.

## Results

### Vascular reactivity studies

**Figure 1:**
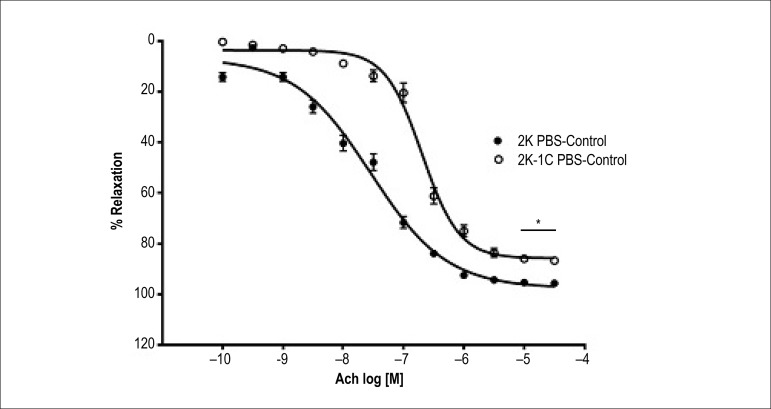
Concentration-response curves (n = 8) for acetylcholine in intact endothelium- aortic rings contracted with phenylephrine. Values are mean ± S.D of experiments performed on preparations obtained from different animals. *** indicates signifcant difference (p < 0.001) in pD2 value for 2K vs. 2K-1C.

As can be seen at Figure 1, acetylcholine induces relaxation in pre-contracted aortic rings. However, the potency and the maximum relaxant effect was lower in aortic rings from hypertensive rats 2K-1C (Tables 1 and 2) when compared to aortic rings of normotensive 2K rats (Tables 1 and 2), indicating endothelial dysfunction in aortic rings of hypertensive rats 2K-1C.

**Table 1:** Potency (pD2) and Maximum relaxant effect (ME) to acetylcholine in endothelium intact aortic rings from 2K and 2K-1C rats incubated with PBS and BPY (0.1*µ*M), and ME to acetylcholine in coronary rings from rats with intact (E+) and denuded (E-) endothelium from 2K and 2K-1C incubated with BPY (0.1*µ*M). Values are mean of *n* experiments performed on preparations obtained from different animals, and number of animals used

		2K-1C	2K
PBS	pD2 Mean; Number of animals (n)	6.34; n = 6	7.07; n = 7
	Emax Mean; Number of animals (n)	71.01, n = 6	93.90; n = 7
BPY 0.1 *µ*M	pD2 Mean; Number of animals (n)	7.74; n= 7	7.32; n = 7
	E_max_ Mean; Number of animals (n)	90.85; n = 6	98.64; n = 7
Intact endothelium (E+)	E_max_ Mean; Number of animals (n)	66.90; n = 5	86.97; n = 5
Denuded endothelium (E-)	E_max_ Mean; Number of animals (n)	34.72; n = 7	34.88; n = 5

**Table 2:** Potency (pD2) and Maximum relaxant effect (ME) to acetylcholine in endothelium intact aortic rings from 2K and 2K-1C rats incubated with PBS and BPY (0.1 *µ*M), and ME to acetylcholine in coronary rings from rats with intact (E+) and denuded (E-) endothelium from 2K and 2K-1C incubated with BPY (0.1 *µ*M). Values are ± S.D of *n* experiments performed on preparations obtained from different animals.

		2K-1C	2K
PBS	Standard Deviation of pD2	± 0.07	± 0.22
	Standard Deviation of E_max_	± 2.58	± 2.79
BPY 0.1 *µ*M	Standard Deviation of pD2	± 0.08	± 0.11
	Standard Deviation of E_max_	± 1.34	± 2.33
Intact endothelium (E+)	Standard Deviation of E_max_	± 2.11	± 5.65
Denuded endothelium (E-)	Standard Deviation of E_max_	± 6.89	± 5.45

**Figure 2:**
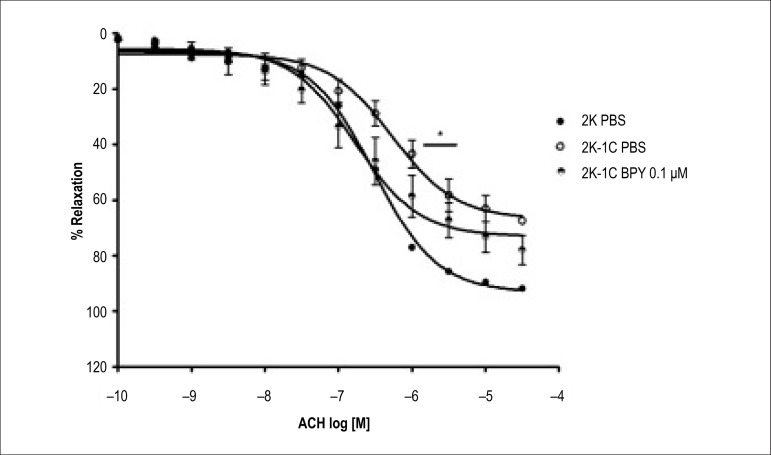
Concentration-response curves for acetylcholine (BPY) in aortic rings with intact endothelium and incubated with different concentrations of cis-[Ru(bpy)_2_(NO_2_-) (NO)](PF_6_)_2_ and contracted with phenylephrine. Values are mean ± S.D of experiments performed on preparations obtained from different animals.* indicates signifcant difference 2K-1C PBS vs 2K-1C BPY 0.1 *µ*M (p < 0.001) e 2K-1C PBS vs 2K PBS (p < 0.001) in pD2.

**Figure 3:**
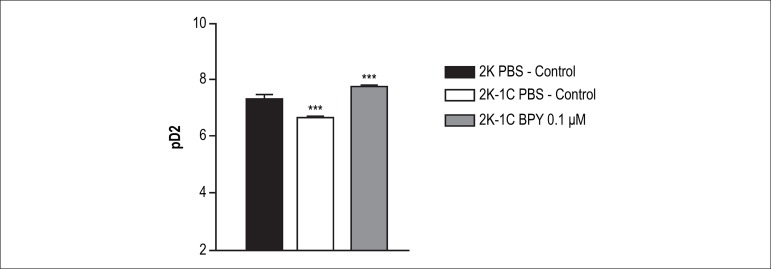
Presents differences in the potency (pD2) of acetylcholine in inducing relaxation in aortas with and without cis-[Ru(bpy)_2_(NO_2_-)(NO)](PF_6_)_2_ treatment. The concentration 0.1 nM normalized relaxation in 2K-1C aortic rings compared to 2K aortic rings. *** - Indicates statistical difference between 2K-1C PBS vs. 2K-1C BPY 0.1 *µ*M (p < 0.001) and 2K-1C PBS vs. 2K PBS (p < 0.001).

Treatment of aortic rings with BPY at 0.1 *µ*M was able to increase the potency of acetylcholine (Ach) in aortic rings of 2K-1C animals (Tables 1 and 2, p < 0.001) when compared with control 2K-1C -PBS (Tables 1 and 2) (Figures 2 and 3).

**Figure 4:**
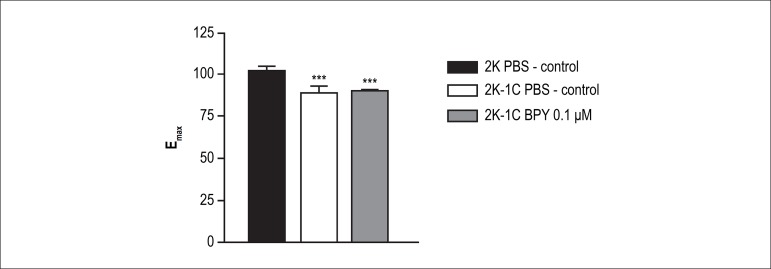
Presents differences in the effciency (E_max_) of acetylcholine in inducing relaxation in aortas with and without cis-[Ru(bpy)_2_(NO_2_-)(NO)](PF_6_)_2_ treatment. The concentration 0.1 nM normalized relaxation in 2K-1C aortic rings compared to 2K aortic rings. *** - Indicates statistical difference between 2K-1C PBS vs. 2K-1C BPY 0.1 *µ*M (p < 0.001) and 2K-1C PBS vs. 2K PBS (p < 0.001).

In addition, the treatment with 0.1 *µ*M of BPY increased the maximum relaxant effect in aortic rings of 2K-1C rats (table 1 and 2, p < 0.001) when compared to the control - 2K-1C PBS (Tables 1 and 2) (Figure 4).

However, the treatment with 0.1*µ*M BPY 2K-1C in aortic rings was able to normalize the potency and the maximum relaxation effect to acetylcholine. In other words, the potency and ME to 2K-1C aortic rings treated with 0.1 *µ*M BPY were similar to that obtained in aortic rings of 2K animals (Tables 1 and 2), suggesting a reversion of endothelial function in 2K-1C aortic ring by treatment with 0.1 *µ*M of BPY (Figures 2, 3 and 4).

**Figure 5:**
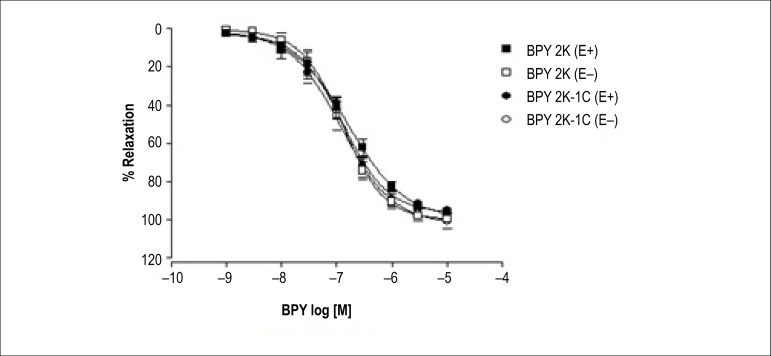
Concentration-response curves for acetylcholine in aortic rings with (E+) and without (E-) intact endothelium, from rats 2K and 2K-1C and incubated with different concentrations of cis-[Ru(bpy)_2_(NO_2_ -)(NO)](PF_6_)_2_ and contracted with phenylephrine. Values are mean ± S.D of experiments performed on preparations obtained from different animals. There was no statistical difference.

As can be seen at Figure 5, the NO donor BPY promoted concentration-dependent relaxation in isolated aortic rings from normotensive (2K) and hypertensive (2K-1C) rats with (E+) and without (E-) endothelium. Moreover, the presence of the endothelium did not change the vasodilating effect induced by BPY compound.

**Figure 6:**
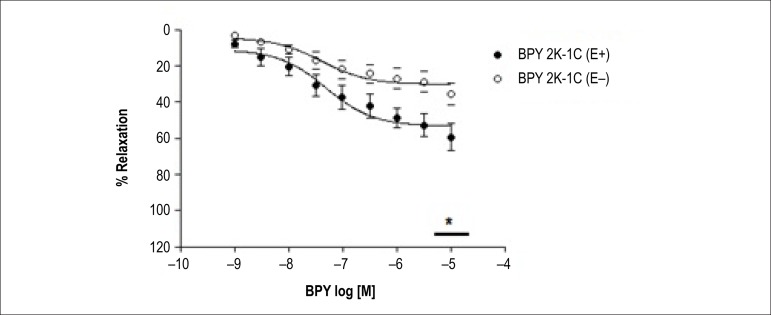
Relaxation coronary artery of rats (2K-1C) with (E +) and without (E-) form endothelium induced by compound cis-[Ru(bpy)_2_(NO_2_^-^)(NO)](PF_6_)_2_ in rings pre-contracted with serotonin (SE). Curves cumulative concentration-effect were performed for BPY compound. Each point represents the mean ± S.D of data obtained from 5-7 independent determinations. * Indicates difference in the value of Emax.

The NO donor *cis*-[Ru(bpy)_2_(NO_2_^-^)(NO)](PF_6_)_2_ (BPY) induced concentration-dependent relaxation in isolated rat coronary with intact (E+) and denuded (E-) endothelium from 2K and 2K-1C animals. As can be seen at figure 6, in coronary arteries of hypertensive (2K-1C) rats, the presence of endothelium potentiated relaxation induced by BPY (Tables 1 and 2) compared to the absence of the endothelium (Tables 1 and 2, p < 0.001).

**Figure 7:**
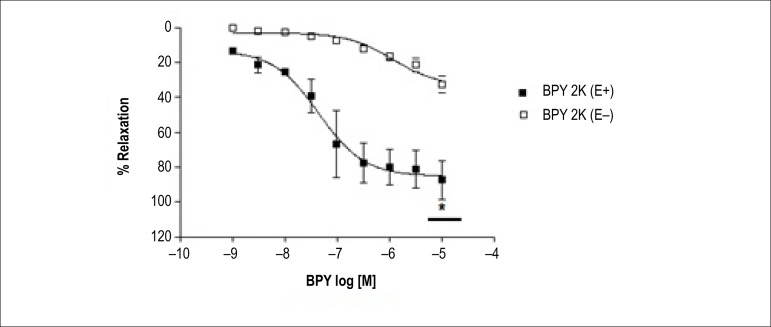
Coronary artery relaxation of normotensive rats (2K) with (E +) and without (E-) form endothelium induced by compound cis-[Ru(bpy)_2_(NO_2_-)(NO)](PF_6_)_2_, in coronary rings contracted with serotonin. Curves cumulative concentration-effect were performed for BPY compound. Each point represents the mean ± S.D of data obtained in fve independent determinations. *Indicates difference in the value of E_max_.

In coronary from normotensive (2K) rats, the endothelium also increased the relaxation induced BPY (Tables 1 and 2, p < 0.001) (Figure 7).

In the absence of the endothelium, BPY compound is able to induce relaxation in coronary from normotensive (2K) rats (Tables 1 and 2) and hypertensive rats (Tables 1 and 2), with no significant difference between the two groups (Figure 7). In intact endothelium coronary arteries, the relaxation induced by BPY was more effective in normotensive animals (Tables 1 and 2) when compared to hypertensive (Tables 1 and 2, p < 0.05) (Figures 6 and 7).

## Discussion

Our results have shown that the endothelium-dependent relaxation induced by acetylcholine is impaired in aortic rings from hypertensive rats (2K-1C). Hypertension model (2K-1C) is mediated by activation of the Renin Angiotensin Aldosterone System, occurring high concentration of circulating Angiotensina II. In accordance with Santeliz et al.,^24^ vascular cells stimulated by angiotensin II show high concentration of superoxide anion (O_2_-) due to activation of NADPH complex, which is responsible for the reduction in the vascular relaxation, since this species produced react with the released NO to form peroxynitrite, thus generating smaller amount of NO available. Furthermore, in hypertensive animals occurs a malfunction in endothelial cell layer due to shear stress and activation of the renin-angiotensin-aldosterone system. This dysfunction is characterized mainly by the decreasing ability of endothelial cells to release NO^1^. The NO produced in the endothelial cell diffuses to a lesser extent into the vascular lumen and for vascular cells smooth muscle^25-28^ causing a failure to control the modulation of vascular tone by NO.

The main finding of the present manuscript was that the treatment with BPY (at concentration 0.1 *µ*M) in hypertensive aortic rings improved the endothelium-dependent relaxation, and was able to normalize the relaxation in 2K-1C aortic rings. These results suggest that a punctual concentration of BPY is able to induce improvement on endothelial function, which could be because of some enzymatic activation or an inhibition generating an increasing effect of endothelium dependent relaxation. It seems that the tonus modulation by endothelial can be improved by BPY.

These results are in accordance with previous study, that have shown an improvement on endothelial function by aortic rings treatment with 0.1*µ*M of another ruthenium compound (*cis-*[Ru(H-dcbpy^-^)_2_(Cl)(NO)]).^4^ Thus, some results have suggested that ruthenium compounds can release NO and improve the endothelial function, which is a desirable effect on vascular system when endothelial dysfunction is present.

The endothelium and hypertension did not change the vasodilator effect induced by BPY compound in aortic rings. Rodrigues et al.,^9^ demonstrated that NO donors, TERPY (ruthenium complex) and SNP as well as BPY promoted concentration-dependent relaxation on isolated aorta from hypertensive (2K-1C) rats and normotensive (2K) rats, without altering the percentage of the maximum relaxation. However the potency of both NO donors (TERPY and SNP) was lower in the aorta from hypertensive rats (2K-1C), different from that observed to BPY, which generated the same potency of relaxation in 2K and 2K-1C aortas. The lower potency to TERPY and SNP was attributed to elevated concentration of O_2_^-^ in aortic rings.^2^ Thus, our results indicate that the vascular effect of BPY is not modified by endothelium or by O_2_^-^ present in aorta 2K-1C.^29^

The endothelium potentiated the relaxation in coronary from normotensive (2K) and hypertensive (2K-1C) rats. This effect was observed just in coronary and not in aorta. In previous study, it was found that the endothelium also potentiated the relaxation induced by SNP in aortic rings,^18^ and we have not found coronary study evaluating the effect of endothelium on relaxation induced by SNP. However, the relaxation induced by BPY is impaired in 2K-1C coronary rings with endothelium, with no difference in the absence. The impaired relaxation is in accordance to our previous study in aortic rings with another ruthenium compound.^2^ but we have not verified any description in coronary. In our opinion, the potentiation of the effect generated on the relaxation was greater in coronary suggesting that in resistance vessels, the endothelium participates in inducing relaxation, and it does not happen in conductance vessels such as the aorta.^30^

## Conclusion

Taken together, our results suggest that 0.1 uM of BPY is able to normalize the endothelium dependent relaxation in hypertensive rats, and the compound BPY induces relaxation in aortic rings from normotensive and hypertensive rats with the same potency. In addition, the endothelium potentiate the relaxation effect induced by BPY in coronary rings from normotensive and hypertensive rats, with lower effect on coronary from hypertensive rats.

### Limitations

The short period of time, corresponding to the duration of a master degree.
